# Protecting the nerve terminals

**DOI:** 10.7554/eLife.35664

**Published:** 2018-03-19

**Authors:** Jonathan D Glass

**Affiliations:** Emory ALS CenterEmory University School of MedicineAtlantaUnited States

**Keywords:** amyotrophic lateral sclerosis, neuromuscular disease, neurodegeneration, motor neuron, agonist antibody, MuSK, Mouse

## Abstract

Maintaining the connections between nerve cells and muscle could help to slow the progression of amyotrophic lateral sclerosis.

**Related research article** Cantor S, Zhang W, Delestrée N, Remédio L, Mentis GZ, Burden SJ. 2018. Preserving neuromuscular synapses in ALS by stimulating MuSK with a therapeutic agonist antibody. *eLife* **7**:e34375. doi: 10.7554/eLife.34375

It is generally accepted in neurobiology that the integrity of the axon – the cable-like extensions that transmit information between neurons, muscles and sensory receptors – is wholly dependent on the health of the neuron cell body. This concept has been a guiding principle in the search for experimental therapeutics to treat neurodegenerative diseases such as Alzheimer’s disease, Parkinson’s disease and amyotrophic lateral sclerosis. In this approach, protecting the cell body of the neuron should help to slow down or even stop the progression of neurodegeneration.

However, growing evidence from experiments in animals and observations in humans has shown that neurons, axons, and even the nerve terminals (which connect to the target muscle) respond differently to damage. This suggests that independent mechanisms of neurodegeneration may be at play in different regions of the neuron (Conforti et al., 2007, 2014). Therefore, treatments may need to target the entire neuron to achieve a significant therapeutic impact.

Amyotrophic lateral sclerosis (ALS) is a devastating motor neuron disease that strikes without warning, typically in the prime of life. People with ALS become progressively weaker, which ruthlessly leads to disability, loss of independence and ultimately death. Therapeutic interventions are largely palliative, and dozens of clinical trials over the past three decades have failed to identify treatments that can slow down – never mind reverse or stop – the progression of this disease.

Promising results from pre-clinical experiments in animal models of the disease have not been successfully translated to humans, and the reasons for these failures are hotly debated. Of particular interest, largely because the findings are paradoxical, have been interventions in animal models of ALS that demonstrated near complete protection of the cell bodies of motor neurons, but failed to slow the progression of the disease. In these cases, protection of the motor neuron cell body by a variety of techniques did not prevent the loss of connectivity between the nerve terminals and the muscle (Gould et al., 2006; Parone et al., 2013; Klivenyi et al., 1999; Suzuki et al., 2007). This loss of connectivity is known as denervation.

Experimental studies in animal models of ALS have shown that the degenerative process of the disease starts at the nerve terminals and progresses proximally to the axon and eventually to the cell body of the neuron, a pattern described as ‘dying-back’ (Frey et al., 2000; Fischer et al., 2004). As with any ‘electrical’ system, the separation of the wire (the axon) from its target (the muscle) results in loss of function – or in the case of ALS, weakness and death. Now, in eLife, Steven Burden and colleagues from NYU Medical School and Columbia University – including Sarah Cantor as first author – report that protecting the connection between nerve and muscle could slow down the progress of the disease (Cantor et al., 2018).

The motor neuron connects to the muscles at the so called neuromuscular junction, through which the neuron transmits signals to activate muscle contraction (Figure 1). Many proteins help to maintain this connection, including one called MuSK (Burden et al., 2013). During development, MuSK stimulates the attachment of the nerve terminal to the muscle. Later in life, MuSK is necessary to stabilize and maintain the neuromuscular junction, which ensures that signals can travel between the muscle, the nerve terminal and the motor axon. Indeed, damage or mutations to the MuSK gene are linked to other neuromuscular diseases.

**Figure 1.**
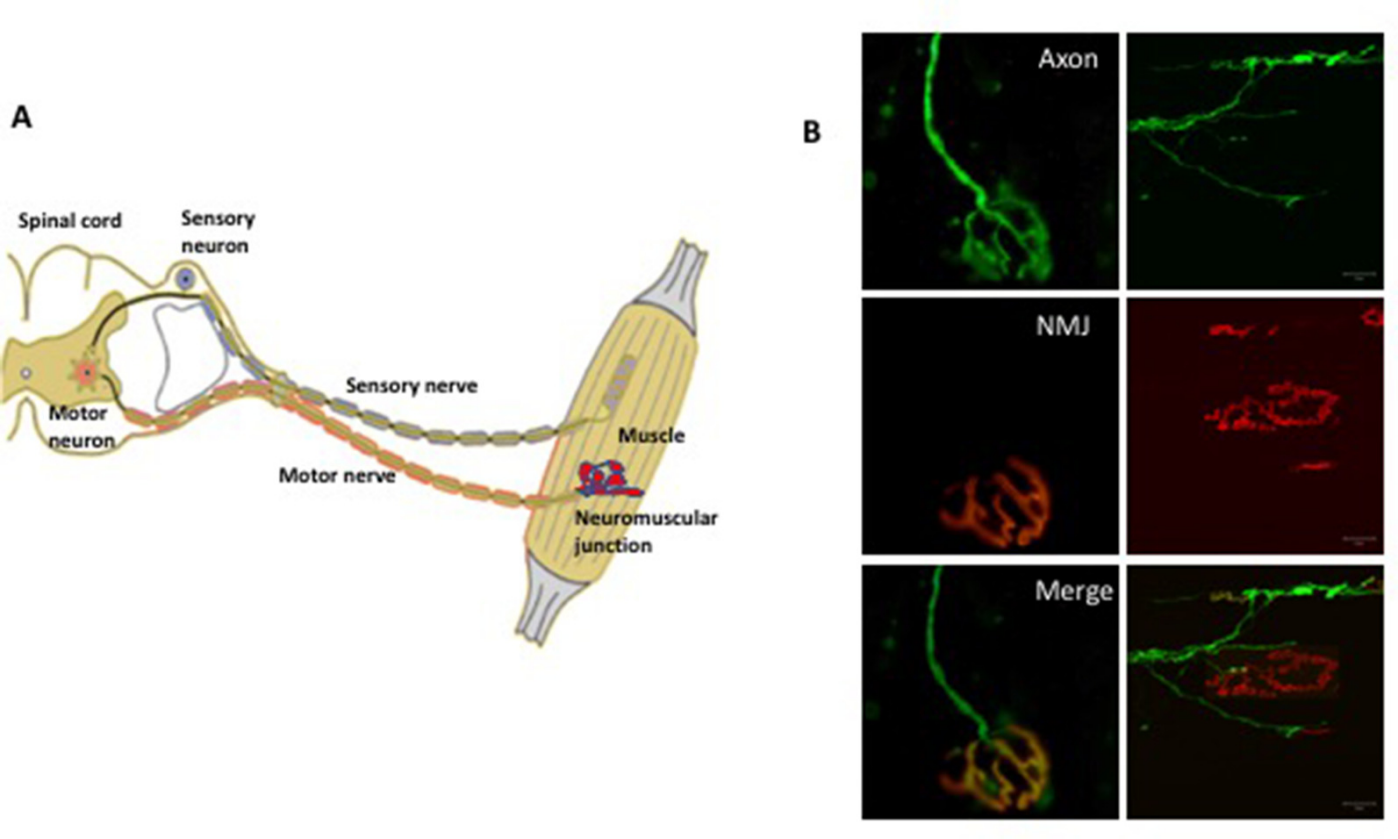
The somatic nervous system. (**A**) A schematic representation of the neuromuscular system. The sensory neurons (shown in blue) convey information (in the form of electrical pulses) from different parts of the body to the brain. Motor neurons (red) in the brain and the spinal cord send electrical pulses along the axons (which form the motor nerve) to the muscle fibers to make them contract. In diseases such as amyotrophic lateral sclerosis, there is evidence that degeneration begins at the neuromuscular junction between the terminals of the axons and the muscle and progresses along the axon towards the cell body. (**B**) Fluorescence microscopy of the neuromuscular junction (NMJ). The panels on the left show a fully innervated neuromuscular junction, with the axon labeled in green (top left) and the muscle labeled in red (middle left). The merged panel (bottom left) shows the complete overlap of the nerve terminal with the muscle (yellow). The panels on the right show a denervated neuromuscular junction in which the axon no longer overlaps with the muscle.

Cantor et al. used a well-characterized mouse model of ALS to test if intervening with already damaged neuromuscular junctions could slow the progression of the disease. Their approach involved using an antibody that binds to and stimulates the activation of MuSK. The results showed that with the antibody, the neuromuscular junctions remained intact and functional for an extended period. In addition, the death of motor neurons was delayed, indicating that maintenance of muscle innervation plays a role in the support of the motor neuron cell body.

But even so, the animals only survived several days longer than normally. This might be due to the aggressive nature of the disease in this animal model, or possibly due to the lack of therapeutic targeting of the motor neuron cell bodies. Nevertheless, these findings suggest that protection of the neuromuscular junction could be a way to slow disease progression and keep neurons alive.

The pathogenesis and progression of ALS is an enigmatic and vexingly unresolved neurobiological problem. Genes and cellular pathways that represent possible upstream drivers of the disease are being discovered on a regular basis, and all of these may be specifically targeted. However, the approach of protecting neuromuscular junctions by targeting the underlying biology of maintenance is agnostic to these upstream mechanisms and is also not specific to ALS. It could therefore help to preserve function in a variety of neuromuscular diseases where denervation is a major pathological feature.
